# Status quo and predictors of Weibo users’ attitudes toward lesbians and gay men in 31 provinces in the Chinese mainland: Analysis based on supervised machine learning and provincial panel data

**DOI:** 10.3389/fpsyg.2023.1069589

**Published:** 2023-02-01

**Authors:** Quan Zheng, Ying Guo, Zhen Wang, Frank Andrasik, Ziyi Kuang, Sheng Xu, Xiangen Hu

**Affiliations:** ^1^School of Psychology, Central China Normal University, Wuhan, China; ^2^Key Laboratory of Adolescent Cyberpsychology and Behavior (CCNU), Ministry of Education, Wuhan, China; ^3^School of Psychology, Sichuan Normal University, Chengdu, China; ^4^School of Psychology, Faculty of Education, Henan University, Kaifeng, China; ^5^Department of Psychology, University of Memphis, Memphis, TN, United States

**Keywords:** consensual same-sex relations, attitude, lesbians and gay men, Chinese mainland, Weibo, social media, natural language processing, machine learning

## Abstract

**Introduction:**

Public attitudes toward consensual same-sex relations are crucial to lesbians’ and gay men’s rights and society’s well-being, but research addressing this topic in China is limited. We comprehensively explored the current status and predictors of Weibo users’ attitudes toward individuals who are lesbian or gay (IWLG) at the provincial level in the Chinese mainland.

**Methods:**

Natural language processing and machine learning techniques were incorporated to analyze 1,934,008 Weibo posts from January 1, 2010, to December 31, 2020, to evaluate Weibo users’ expressed attitudes toward IWLG in 31 provinces in the Chinese mainland guided by the ABC Model of attitude.

**Results:**

Although the general attitudes, feelings, and support for the rights of Weibo users toward IWLG among different provinces were relatively positive, knowledge about IWLG was noticeably inaccurate. Economic development and educational level positively predicted certain aspects of attitudes at the provincial level.

**Conclusion:**

Weibo users from different provinces are generally supportive and accepting of people who are gay and the rights of the gay community. However, considerable misconceptions and inaccurate knowledge of IWLG surfaced in Weibo users’ posts. Economic development and educational level were important predictors of specific attitudes toward IWLG at the provincial level. Increased efforts to address the unbalanced and insufficient development between different provinces could help reduce the public’s prejudice, stigma, and discrimination toward IWLG. Policies that facilitate greater implementation of Comprehensive Sexuality Education sequentially and effectively are suggested as well.

## 1. Introduction

Consensual same-sex relations (CSSR)^1^ are natural, normal, and recognized as one of the three main orientations in human sexuality (American Psychological Association, 2008; Pan American Health Organization, 2012). Despite progress in acceptance and recognition of LGBTQ+ people around the world, this progress is vulnerable and varies significantly across different countries (Salvati and Koc, 2022). Even though CSSR was decriminalized in 1997 and de-medicalized in 2001 in the Chinese mainland (Xie and Peng, 2018), discrimination and stigmatization against individuals who are lesbian or gay (IWLG) appear to persist to a large extent in Chinese society (Miles-Johnson and Wang, 2018; Ren et al., 2019). The continued stigma and discrimination harm not only the IWLG but society as well (Zheng et al., 2022). Gaining a deeper understanding of public attitudes toward IWLG that can help guide development of policies and measures to maximize the effectiveness of actions to diminish and over time hopefully eradicate public discrimination against IWLG from a specific social psychological perspective is urgently needed and very important in contemporary Chinese society.

Research focussing on particular locations (e.g., Beijing) or groups of select individuals (e.g., college students) have identified certain individual factors (e.g., gender, age, education level) that influence the attitudes toward IWLG in the Chinese mainland (Lin et al., 2016; Xie and Peng, 2018; Hu and Li, 2019; Wang et al., 2020; Zhou and Hu, 2020; Lin and Wang, 2021; Liang et al., 2022; Zheng et al., 2022). However, China is a country that has 34 provincial-level administrative divisions, which vary significantly with respect to culture, economic development, and public opinion. Consequently, it is not surprising that the available research has identified a wide variability in attitudes toward IWLG among different populations in different administrative divisions in China. For example, Qu et al. (2015) found that about 70% of college students in Yunnan province believed that consensual same-sex sexual activity was abnormal. However, Lin et al. (2016) found that Chinese college students in the municipality of Beijing and Fujian province generally held accepting attitudes toward IWLG. These divergent results highlight the critical importance of investigating the public attitudes toward IWLG at the provincial level to gain a more comprehensive understanding of Chinese public opinion about IWLG and to help identify the most effective ways to tailor approaches to diminish public discrimination against IWLG in different administrative divisions in China. Although some research has investigated Chinese public attitudes about IWLG at the provincial level (Chua et al., 2019; Wang et al., 2020; Zhang et al., 2020), this research is limited in sample size and scope, with most addressing a single aspect of attitude (e.g., general acceptance), which raises concerns about the generalizability of the reported results. Moreover, obtaining reliable attitudes toward IWLG at the provincial level using conventional methods, such as surveys, can be difficult due to various challenges, including generating a sufficient sample size while maintaining cost and time constraints. According to Salvati and Koc (2022), one of the key directions for research on the social psychology of sexual orientations and gender identities is to utilize a diverse range of methodologies, including qualitative and mixed-methods approaches, to more fully understand the complexity of experiences and attitudes related to these topics. By doing so, researchers could better capture the range of experiences and attitudes related to sexual orientations and gender identities.

Natural Language Processing (NLP) and Machine Learning (ML) techniques are effective methods for addressing and achieving the limitations and goals as mentioned earlier, respectively, of this field of research (Zheng et al., 2022). They can do this by unobtrusively and accurately analyzing large amounts of human behavior data that is recorded constantly and non-intrusively on the Internet. Using these techniques could allow researchers to gain a more thorough and accurate understanding of the attitudes toward IWLG at the macro level, which is the main focus of this study. In this study, we adapted these approaches to analyze IWLG text data downloaded from Sina Weibo, one of the largest and most influential social media platforms in China, to explore Chinese public attitudes toward IWLG at the provincial level based on the ABC model of attitude. We additionally focused on identifying specific factors that might potentially influence the attitudes toward IWLG, with respect to provincial-level variables (e.g., economic development and educational level), to enable us to gain a more comprehensive understanding of the attitudes toward IWLG and provide targeted suggestions to promote the acceptance of IWLG in the Chinese mainland.

## 2. Literature review and current study

### 2.1. Provincial-level variables that may influence the attitudes toward IWLG

As mentioned previously, much research has been devoted to understanding the impact of individual characteristics on attitudes toward IWLG in the Chinese mainland. However, it is also important to examine the role of provincial or macro-level factors in shaping these attitudes. Research has revealed that macro-level factors, including economic development, educational level, religion, and politics, play a significant role in shaping attitudes toward IWLG (Adamczyk and Liao, 2019; Badgett et al., 2019; Bettinsoli et al., 2020; Zhou and Hu, 2020). In this study, we are focusing on economic development and educational level at the provincial level as these factors are deemed significant and can be accurately measured.

#### 2.1.1. Economic development

Economic development may have significant influences on attitudes toward IWLG. Research supporting modernization theory has identified the important connection between economic growth and value shifting (Inglehart, 1971), including changes in perceptions of IWLG-related issues (Gerhards, 2010). On average, residents within wealthier countries tend to have more positive attitudes toward sexual minorities than do individuals residing within poorer ones (Hadler, 2012). Wang et al. (2020) found a significant negative correlation between the level of economic development (GDP *per capita*) in provinces and discrimination against sexual minority individuals in the Chinese mainland. However, findings from these studies are by no means definitive as the sample sizes were quite small. We attempted to address this shortcoming by systematically extracting more extensive data regarding attitudes toward IWLG from Weibo.

#### 2.1.2. Educational level

Multiple studies have identified the essential function of education in forming attitudes toward IWLG at the individual level. Ohlander et al. (2005) argue that education elevates the tolerance of IWLG by teaching people to be more accepting of nonconformity and promoting greater cognitive complexity and complex reasoning to better evaluate different ideas. Some research has found that a higher level of education is usually associated with increased acceptance of IWLG (Xie and Peng, 2018; Zhou and Hu, 2020; Zheng et al., 2022). Although China has made significant progress in increasing national educational level and reducing illiteracy, education development remains inconsistent and unequal across provinces. The varied levels of educational level across provinces are likely to result in different degrees of tolerance toward IWLG as well. To our knowledge, research on the effects of education on attitudes toward IWLG mainly focuses on the individual level rather than the province level, with us exploring this as well in the present study.

### 2.2. Theoretical perspective

Most of the research on attitudes toward IWLG discussed above has mainly targeted a single aspect (such as support for equality) or a general attitude. However, the ABC model of attitude (also known as the tripartite model of attitude), a well-established theory that has been widely accepted and supported by research in the field of psychology (Eagly and Chaiken, 1993; Liu et al., 2021), highlights the importance of remembering that an attitude has three important components: affect, behavior, and cognition (Rosenberg and Hovland, 1960; Breckler, 1984; Manstead, 1996). Each of these components is important to consider when studying attitudes (Zheng et al., 2022). The affect component refers to the emotional or feeling component of an attitude. It includes the feelings and emotions that an individual has toward a particular object or issue. The behavior component of an attitude refers to the way an individual behaves or tends to behave toward a particular object or issue. It includes actions and behaviors, or behavioral tendencies, that a person engages in due to their attitudes. The cognition component refers to the thoughts and beliefs that an individual holds about a particular object or issue. It includes the knowledge and information that an individual has about an object or issue.

Using the ABC model of attitude as a guide, we attempted to evaluate attitudes toward IWLG at the province level more thoroughly and explicitly than typically done in the past. In order to accomplish this, we began our approach under the assumption that text posted on Weibo is composed of a comparatively enduring structure of beliefs, emotions, and behavioral tendencies that Weibo users hold with respect to IWLG (Zheng et al., 2022). We assumed further that expressed comments would have the following three components: (1) An affect component that includes Weibo users’ feelings/emotions about IWLG. (2) A behavioral component that involves the behavior or behavioral tendencies Weibo users have about IWLG, consisting of whether they: (a) are willing to maintain friendship or collegiality with IWLG, (b) support the legalization of marriage or partnership for same-sex couples, and (c) hold opinions that protect the rights of IWLG. (3) A cognition component that involves the beliefs or knowledge of Weibo users about IWLG.

### 2.3. The current study and overview

The current study was designed to explore the current state of Weibo users’ attitudes toward IWLG across different provinces in China and begin to identify potential provincial indicators that may influence the Chinese public attitudes toward IWLG at the provincial level based on large-scale text data gathered from Weibo. Our approach incorporated NLP and ML techniques to analyze Weibo text related to IWLG attitudes across different provinces in China based on the ABC model (affect, behavior, and cognition components) as well as general attitudes. Specifically, this study attempted to address the following questions: (1) What is the current state of Weibo users’ attitudes (three components and whole) toward IWLG across different provinces in the Chinese mainland?, (2) What province-level variables (e.g., economic development, educational level) might best predict Weibo users’ attitudes toward IWLG at the provincial level?

## 3. Methods

**Figure 1:**
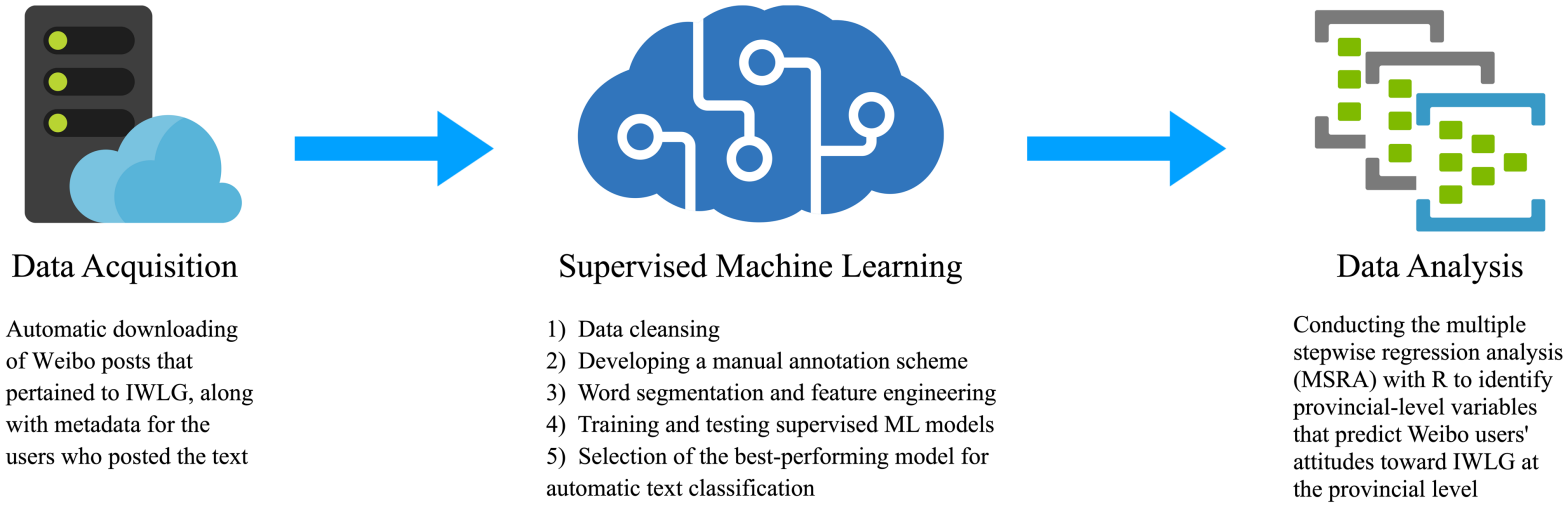
Methodology for data acquisition and analysis.

We adopted the research methodology used by Zheng et al. (2022) as the foundation for our study. However, we made certain modifications and additions to the methodology in order to more specifically address the research questions and objectives of our study. These modifications included adding data from 2020 and conducting additional analyses to examine the relationship between provincial-level variables and attitudes toward IWLG, and to observe differences between provinces. By building upon the established methodology of Zheng et al. (2022) and making these modifications, we were able to gain a more comprehensive understanding of the topic. The structural methodology schema of the data acquisition and analysis is illustrated in Figure 1. A more precise representation of each main step (data acquisition, supervised machine learning, and data analysis) is provided in subsequent sections.

### 3.1. Data

The present study used the Chinese word “同性恋” (which refers to IWLG in English) as the keyword and searched for posts containing this keyword every day from January 1, 2010, to December 31, 2020, through the Weibo advanced search engine. A web crawler was used to automatically download all of the target posts and metadata (including the self-reported location) of Weibo users. All data downloaded from Weibo is publicly available and accessible to anyone with a Weibo account.

2,238,239 Weibo posts pertaining to IWLG appeared during the data collection period, with 1,934,008 posts available for analysis after data cleansing (i.e., removing ads, messy codes, etc.).

### 3.2. Variables

The dependent variable was Weibo users’ exposed attitudes toward IWLG at the provincial level. Each Weibo post concerning IWLG was assessed and rated with respect to the four dimensions in the ABC Model as previously discussed (affect, behavior, and cognition components, along with the general attitude).

Each post was independently assessed and assigned a score value, ranging from 0 to 5, where 0 = “invalid,” which indicated that the post did not concern the distinct component. For the affect and behavior components, 1 = “strongly negative,” 2 = “somewhat negative,” 3 = “neutral,” 4 = “somewhat positive,” and 5 = “strongly positive.” For the cognition component, 1 = “completely inaccurate,” 2 = “somewhat inaccurate,” 3 = “neutral,” 4 = “somewhat accurate,” and 5 = “completely accurate.” The general attitude score consisted of the average value of the three separate components. The criteria for rating scores for the cognition component have been based on the agreement^2^ gained in the most current CSSR study (American Psychological Association, 2008; Pan American Health Organization, 2012).

Various independent variables were obtained from each of the 31 provinces^3^ in the Chinese mainland in order to examine differences due to the following two dimensions: (a) *Economic Development*—Although GDP *per capita* is an ideal indicator of the economic development of each province in the Chinese mainland, it is highly correlated with educational level (*r* = 0.64, *p* < 0.001). In the current study, we opted to use the GDP (another commonly used economic development indicator) in 2020 (National Bureau of Statistics of China, 2021) as the optimal indicator of the economic development of each province to avoid the bias of multicollinearity. (b) *Educational level*—The Education Index (a composite measure of adult literacy rate and combined primary, secondary, and tertiary enrollment rates), released by the United Nations Development Programme (UNDP), Tsinghua University, and State Information Center (2019), was selected as the best measure of the educational level for each province.

### 3.3. Natural language processing and supervised machine learning

#### 3.3.1. Manual annotation

Manually handling the massive amount of Weibo text data that needed to be processed and analyzed was not feasible. In situations such as this, supervised ML and NLP techniques have been used successfully and efficiently to mine the variables of interest from Weibo text data. Before conducting supervised ML, annotated instructional data are used to train and test various classification models.

In order to accomplish this, one author manually coded a sample of 39,986 Weibo posts, which were randomly extracted by day from all Weibo posts from 2010 to 2020 (~10 texts per day) based on stratified random sampling to maintain the representation of the annotated data. The methods outlined above were used to rate all attitude components. For example, “同性恋不仅违反世俗，还违反自然规律。违自然者，可悲，必亡。” (CSSR is not only against the common customs but also against the laws of nature. The one who goes against nature is pathetic and will perish.) This text was coded as 1, 0, and 1 for affect, behavior, and cognition components. It shows strong negative feelings or emotions, completely inaccurate beliefs or knowledge about CSSR, and does not involve the behavioral component. The second example is, “总有一天歧视将不复存在。同性恋也好，异性恋也罢，纯真的爱情不应该受到歧视，而应该是被祝福的。” (One day, discrimination will cease to exist. Either innocent love between same-sex or heterosexuals should not be discriminated against, but blessed!) This text was coded as 5, 5, 5 for affect, behavior, and cognition components because it reveals a solid optimistic trait on these three components. The last example is, “我想问大家一句。你们对同性恋怎么看啊?” (I would like to ask everyone a question. What is your opinion about CSSR?) Text like this was coded as 0, 0, 0 for each component because we lacked enough information to make an assessment.

To further evaluate the manual annotation’s reliability, 1,000 texts were randomly selected from the aforementioned 39,986 posts in the corpus and were independently annotated by two different authors before the formal manual annotation was begun (Zheng et al., 2022). Following the guidance of Hubley and Tremblay (2002), we used the Pearson correlation and intraclass correlation (ICC) to measure the agreement between the two coders. We used the single-measure, two-way mixed effects, and absolute agreement methods, with ICC values between.75 and 1.00 indicating excellent agreement (Cicchetti, 1994). Our results showed that the levels of agreement for the affect (Pearson’s *r* = 0.83, ICC = 0.82), behavior (Pearson’s *r* = 0.84, ICC = 0.80), and cognition (Pearson’s *r* = 0.82, ICC = 0.78) components were all within the acceptable range, as determined by the guidelines established by Cicchetti (1994). Discrepancies were resolved through discussions, with the scoring criteria updated as needed to reflect the concurred perspectives. Finally, one of the two authors formally rated the remaining 38,986 posts. Table 1 summarizes the details of the manual annotation.

**Table 1:** Manual annotation of training and testing text and performance of linear SVMs model.

Components *N*	Score	Annotation text *N*	Model evaluation				
			Accuracy	Precision	Recall	F1	10-fold CV F1
Affect 34,142	1	5,112	0.74	0.74	0.74	0.74	0.74
	2	4,551	0.68	0.68	0.68	0.68	0.69
	3	2,878	0.65	0.65	0.65	0.65	0.65
	4	3,543	0.68	0.68	0.68	0.68	0.67
	5	18,058	0.80	0.80	0.80	0.80	0.79
Behavior 18,107	1	3,731	0.75	0.75	0.75	0.75	0.75
	2	914	0.75	0.75	0.75	0.75	0.72
	3	518	0.74	0.75	0.74	0.74	0.75
	4	586	0.68	0.68	0.68	0.67	0.68
	5	12,358	0.84	0.84	0.84	0.84	0.84
Cognition 3,159	1	1,174	0.78	0.78	0.78	0.78	0.76
	2	178	0.76	0.76	0.77	0.77	0.71
	3	171	0.70	0.70	0.71	0.70	0.74
	4	361	0.72	0.72	0.72	0.72	0.78
	5	1,275	0.85	0.85	0.85	0.85	0.85

#### 3.3.2. Word segmentation and feature engineering

For this process, we built a python program with a popular Chinese word segmentation tool, the Jieba package^4^. For each input text data, the tokenization process splits the text string into individual words and omits the stop words (a stop word is a commonly used word, such as “a” and “the” that does not add much meaning to a sentence).

Feature engineering was implemented to convert Weibo posts into a format that a computer can recognize through series processing. We adopted the vector space model to express the features of the Weibo post and the TF-IDF (Term Frequency – Inverse Document Frequency) as the specific feature extraction index.

After the word segmentation process was completed, it was then possible to count the words in the corpus and retain them as features. Determination of the discriminant words takes into account the frequency of the words in a single document (one particular post in text data; DF) and the scarcity of the words in all documents (IDF). We adopted the above-mentioned TF-IDF (Ramos, 2003), the product of TF and IDF, to measure the importance of vocabulary. The features of each Weibo post could then be represented as a feature vector, with each dimension being equivalent to a specific word. This feature vector for each input Weibo post could be created based on the words of this post by the program we constructed, which was utilized as the input for various ML models.

#### 3.3.3. Training and testing supervised ML models

The goal of supervised ML in this study was to fit a variety of binary classifiers to the annotated dataset and then apply them to predict attitudinal scores for the remaining Weibo texts. We tested the following candidate classification models to ascertain the best-performing one: (1) k-nearest neighbors (KNN), (2) decision trees (DT), (3) random forests (RF), (4) gradient boosting trees (GBT), (5) linear support vector machines (linear SVMs), (6) Naive Bayesian (NB), and (7) logistic regression (LR). The following metrics were selected to evaluate the performance of each candidate ML model: accuracy, precision, recall, and F1. Moreover, the 10-fold cross-validation (CV; Kohavi, 1995) was adopted to obtain a more robust evaluation.

#### 3.3.4. Best-performing model selection and automatic text classification

To implement ML, we built a Python program with the Scikit-learn package (Pedregosa et al., 2011). In general, the linear SVMs model performed better than the other candidate models, obtaining acceptable accuracy, precision, recall, and F1 values. Table 1 summarizes the performance of the linear SVMs model for each attitudinal aspect. Given the overall sound performance of the linear SVMs model, we adopted this model for automatic text classification.

The best-performing model for each attitudinal component was used to automatically predict the classification of all unannotated Weibo posts, all of which received scores ranging from 0 to 5 for the affect, behavior, and cognition components.

### 3.4. Data analysis

All data analyses were conducted with R (Version 4.0.0) and RStudio (Version 1.2.5042), using the “readr” (Wickham et al., 2020), “psych” (Revelle, 2020), “Hmisc” (Harrell, 2021), and “QuantPsyc” (Fletcher, 2012) packages. Multiple stepwise regression analysis (MSRA) was used to explore the impact of provincial-level variables on Weibo users’ attitudes toward IWLG at the provincial level. Pearson correlation coefficients were calculated to explore the potential associations among all variables included in the study.

## 4. Results

After automatic text classification, we excluded 44,214 Weibo text data that did not address any of the target attitudes (those scored as 0) and 798,383 Weibo text data wherein the users did not identify themselves as being located within 1 of the 31 provinces of the Chinese mainland. This left us with 1,091,411 valid data points for statistical analysis. We additionally used the average score of Weibo users (excluding any posts scored as 0) to express the score of each component and adopted the average score of each component as the overall attitude score.

### 4.1. General attitudes expressed across the 31 provinces

Table 2 summarizes the findings regarding Weibo users’ overall attitudes toward IWLG across the 31 provinces in the Chinese mainland. The attitude scores were quite similar, ranging from 2.73 to 3.36, across the four attitudinal aspects. The cognition component was determined as somewhat inaccurate (<3.00), with the remaining attitudinal aspects being evaluated as relatively positive (>3.00). Among all three attitude components, the affect component accounted for the largest proportion of Weibo users’ expressions about IWLG.

**Table 2:** The overall findings on four attitudinal aspects of Weibo users toward IWLG across 31 provinces.

Components	Text data	*M* (SD)	Percentage (%)
Affect	10,17,726	3.36 (1.68)	93.25
Behavior	714,954	3.28 (1.60)	65.51
Cognition	711,358	2.73 (1.50)	65.18
General		3.12 (1.59)	

### 4.2. Weibo users’ attitudes toward IWLG at the provincial level

**Figure 2:**
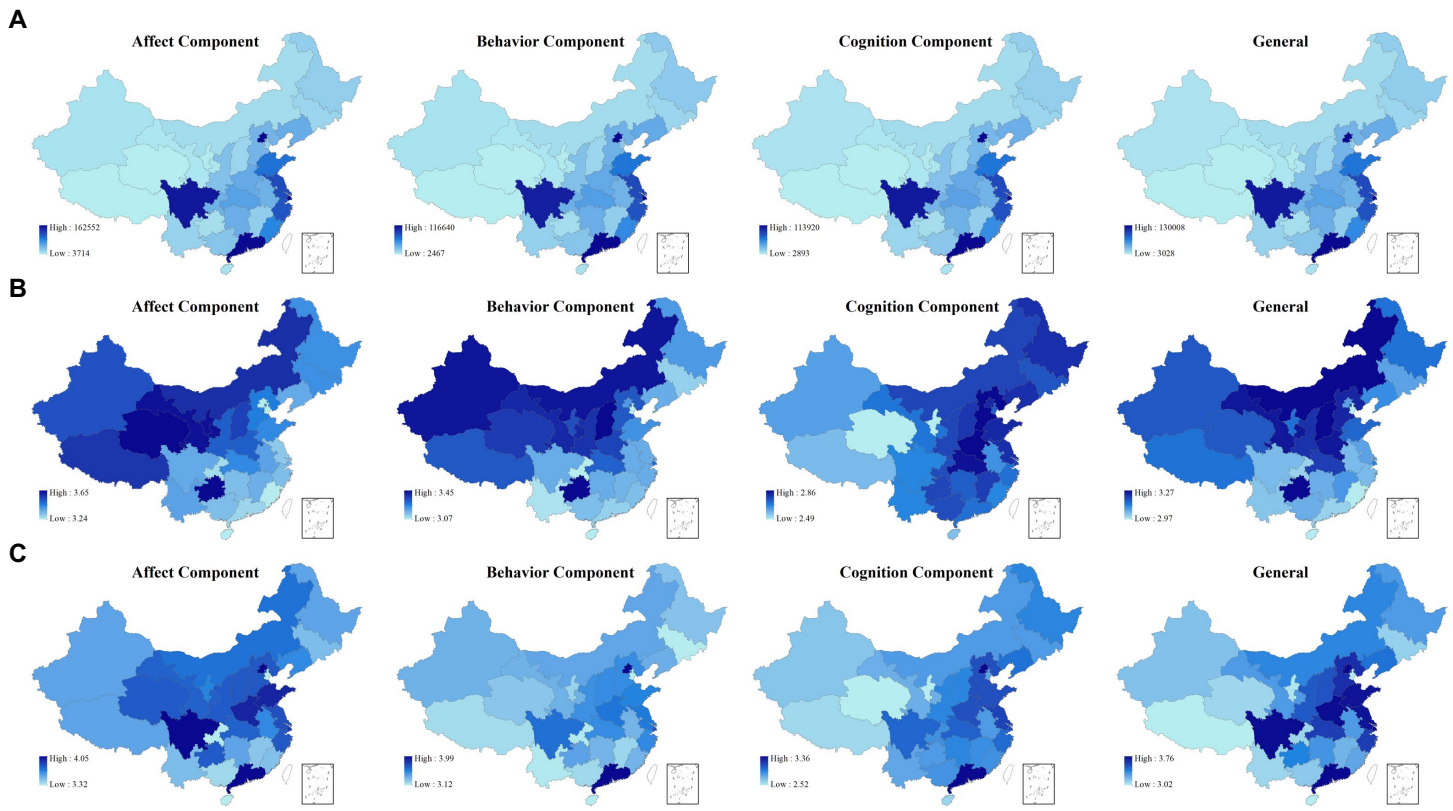
Choropleth maps: **(A)** Geographical distribution of Weibo text related to four aspects of Weibo users’ attitudes toward IWLG among 31 Chinese mainland provinces. **(B)** Geographical distribution of attitudinal score of four aspects of Weibo users’ attitudes toward IWLG among 31 Chinese mainland provinces. **(C)** Geographical distribution of adjusted attitudinal score of four aspects of Weibo users’ attitudes toward IWLG among 31 Chinese mainland provinces.

We next calculated the Weibo post numbers and the average attitudinal scores of each attitude component among the 31 provinces in the Chinese mainland based on the location reported by Weibo users to present the Weibo users’ attitudes toward IWLG at the provincial level. The resultant descriptive statistical findings are presented in various groupings in Figure 2 (see Supplementary material for detailed data of Figure 2).

#### 4.2.1. Geographical distribution of text number of attitudes among 31 provinces

As shown in Figure 2A (the first row of Figure 2), across the 31 provinces in the Chinese mainland, for general, the largest and smallest five provinces in terms of Weibo post numbers were Beijing (130,008), Guangdong (129,347), Shanghai (70,038), Sichuan (58,389), Jiangsu (46,037), and Tibet (3,028), Qinghai (3,111), Ningxia (3,665), Gansu (5,469), Xinjiang (5,804), respectively. When controlling for the effect of the total resident population in each province, the Pearson correlation coefficient between the GDP of the province and the number of Weibo posts was statistically significant (0.63, *p* < 0.001).

#### 4.2.2. Geographical distribution of attitudinal score among 31 provinces

Figure 2B (the second row of Figure 2) shows the score of Weibo users’ attitudes toward IWLG for each province on the three separate, as well as the general components. In general, Weibo users from the western provinces^5^ with fewer Weibo posts revealed more positive attitudes toward IWLG. Moderate negative Pearson correlation coefficients of −0.48 (*p* < 0.01) and −0.42 (*p* < 0.05) were found between the affect scores of each province and the number of Weibo posts and GDP, respectively. Taking into account the geographical distribution of Weibo users (as the more economically developed eastern coastal provinces have a greater number of users, while the western inland areas have fewer users) and the particularity of CSSR issues in the Chinese mainland (CSSR issues are less discussed among people in economically underdeveloped regions), Weibo users’ attitudes in western provinces (e.g., Qinghai, Guizhou, Shanxi, Gansu) were observed to be more favorable toward IWLG, which may indicate that sampling errors existed among the attitudinal scores of these provinces. In order to facilitate comparison with other national survey studies, the current study weighted the attitudinal scores of Weibo users in each province with the proportion of posts and the proportion of the Baidu Search Index of the Chinese keyword “同性恋”^6^ (Baidu, 2021) in each province. The adjusted score was thus equal to the original score combined with the product of the original score and the sum of the proportion of posts and the search index proportion of each province.

#### 4.2.3. Geographical distribution of adjusted attitudinal score among 31 provinces

The adjusted attitudinal scores of each province on the three separate and the general components are shown in Figure 2C (the third row of Figure 2). A relatively positive attitude was observed toward IWLG among Weibo users (>3.00) across the 31 provinces in the Chinese mainland for all components except for cognition. The top five provinces with relatively positive expressions of feelings or emotions (> 3.00) were found with respect to the affective component in Guangdong (4.05), Beijing (3.95), Sichuan (3.82), Henan (3.78), Shandong (3.78). The top five provinces with relatively higher levels of support (> 3.00) for the behavioral component surfacing in Beijing (3.99), Guangdong (3.94), Shanghai (3.75), Sichuan (3.68), Henan (3.66), Jiangsu (3.65). The top five provinces with relatively accurate beliefs about IWLG (> 3.00) were found with respect to the cognition component in Beijing (3.35), Guangdong (3.29), Jiangsu (3.10), Shanghai (3.07), and Hebei (3.06). Finally, the five following provinces revealed the highest general attitude scores: Beijing (3.76), Guangdong (3.76), Shanghai (3.52), Sichuan (3.50), and Henan (3.50).

#### 4.2.4. Predictors of Weibo users’ attitudes toward IWLG at the provincial level

Table 3 shows the Pearson correlation coefficient matrix between Weibo users’ attitudes for each province and its economic development and educational level. Significant moderate to strong correlations were observed between the three separate components and general attitude and most of the provincial-level independent variables.

**Table 3:** Correlation matrix of dependent variables and provincial-level independent variables.

	1	2	3	4	5	6
1 Affect component	–					
2 Behavior component	0.94^***^	–				
3 Cognition component	0.68^***^	0.83^***^	–			
4 General	0.92^***^	0.98^***^	0.90^***^	–		
5 Economic development	0.59^***^	0.65^***^	0.76^***^	0.72^***^	–	
6 Educational level	0.20	0.39^*^	0.56^**^	0.42^*^	0.22	–

*p < 0.05; **p < 0.01; ***p < 0.001.

Multiple stepwise regression analysis (MSRA) was performed to explore the impact of provincial-level variables on Weibo users’ attitudes toward IWLG at the provincial level. Table 4 shows the results of the MSRA for the three separate attitudinal models and the general score model with respect to economic development and educational level at the provincial level. The affect model revealed that economic development significantly and positively influenced the feelings/emotions of Weibo users toward IWLG at the provincial level. The behavior model indicated that economic development positively and significantly influenced the support for the equality for gay people of Weibo users toward IWLG at the provincial level. The cognition model showed the economic development and educational level of each province positively and significantly influenced the Weibo users’ knowledge about the IWLG of that province. In the general score model, both economic development and educational level were associated with the general score of Weibo users’ attitudes at the provincial level.

**Table 4:** Multiple regression models predict Weibo users’ attitudes at the provincial level.

	Affect model β (SE)	Behavior model β (SE)	Cognition model β (SE)	General score model β (SE)
Economic development	0.59^***^ (0.01)	0.65^***^ (0.01)	0.66^***^ (0.01)	0.66^***^ (0.01)
Educational level	0.08 (0.31)	0.25 (0.32)	0.41^***^ (0.25)	0.28^*^ (0.26)
$R_{adj}^{2}$	0.33	0.41	0.72	0.56
*F* value	15.79^***^	21.63^***^	38.60^***^	20.28^***^
*N*	31	31	31	31

Although educational level was not included in the affect and behavior model when the multiple stepwise regression analysis was conducted, we have included the regression coefficients for educational level and affective and behavioral attitudes in these two models to increase transparency and interpretability. *p < 0.05; **p < 0.01; ***p < 0.001.

## 5. Discussion

Regarding research question 1, although the general attitude, feelings, and support for equality of Weibo users toward IWLG among different provinces were relatively positive, the knowledge of Weibo users about IWLG was relatively inaccurate. Generally speaking, Weibo users in coastal provinces among Southeast China held more accepting attitudes toward IWLG than users in inland provinces. For research question 2, we found that economic development and educational level significantly and positively predicted some aspects of the attitude at the provincial level.

### 5.1. Economic development and Weibo users’ attitudes toward IWLG at the provincial level

Consistent with the findings of Wang et al. (2020), our findings revealed that economic development might play an important role in shaping attitudes toward IWLG. Our study also found that the impact of economic development on attitudes toward IWLG varies among provinces in the Chinese mainland. For example, provinces with higher levels of economic development tended to have more accepting attitudes toward IWLG compared to provinces with lower levels of economic development. These findings supported the tenants of modernization theory.

According to modernization theory, the level of wealth and economic development in a region can influence the values and priorities of its residents (Inglehart, 1971; Inglehart and Baker, 2000). As a region becomes more affluent, its citizens may place greater emphasis on personal freedom, quality of life, and tolerance for individual differences. In contrast, in areas facing poverty and economic challenges, survival concerns and reliance on close-knit social groups may be more prominent. This may help partially account for our finding that the effect of economic development on attitudes toward IWLG was reflected not only in the general attitude but also in the three attitudinal components among different provinces. China has achieved extraordinary economic development since the Reform and Opening-up in 1978. Along with this continuously deepening process, the powerful forces of modernization, globalization, and Westernization have had a tremendous impact on Chinese society, especially in the coastal provinces, which were the first to implement the Reform and Opening-up. As these provinces have become more and more open, the publics’ living standards are improving continuously, and the ways of people’s lives are diversifying. Correspondingly, these changes have led Chinese cultural values to transform from traditional (e.g., obedience to authority, male superiority) to modernity (e.g., equal rights, independence; Cai et al., 2020) and reshape Chinese attitudes toward various social issues (Xie and Peng, 2018).

Along with these trends, the Chinese central government also set the stage for some positive transitions in attitudes toward IWLG. For example, even the *People’s Daily*, the Chinese Communist Party’s official newspaper, has generally reported IWLG-related news/issues with more positive and less discriminatory and prejudiced media representations in recent years (Huang, 2018). In addition, issues pertinent to LG have been presented more positively in the official English-language media of China and to global audiences (Wang and Ma, 2021).

Meanwhile, higher economic development in a province helps occasion higher levels of infrastructure, educational level, living standards, modernization, and security, which may contribute to higher levels of tolerance toward IWLG. For example, Ayoub and Garretson (2017) found that increased Internet access (in conjunction with higher levels of infrastructure) may contribute to the growing support for sexual minorities globally. Additionally, research by Xie and Peng (2018) suggests that factors commonly associated with modernity, such as education, exposure to Internet information, and liberal inclinations, tend to predict higher levels of tolerance toward IWLG. These positive economic development outcomes may play an essential role in contributing to increased social tolerance of IWLG at the provincial level.

### 5.2. Educational level and Weibo users’ attitudes toward IWLG at the provincial level

As mentioned, extensive research has confirmed that higher levels of education generally result in higher levels of acceptance of IWLG at the individual level (Herek, 1984; Ohlander et al., 2005; Jäckle and Wenzelburger, 2015; Zheng et al., 2022). However, our study found that at the provincial level, the relationship between education and attitudes toward IWLG is more complex. To gain a deeper understanding of this relationship, we analyzed the impact of educational level on various aspects of attitudes toward IWLG among Weibo users at the provincial level, including affective responses, behavioral tendencies, cognitive components, and overall attitudes. We found that educational level was positively correlated with knowledge about IWLG, but did not significantly impact feelings, emotions, or behavioral tendencies toward IWLG across provinces.

This may be because the feelings, emotions, and support for equality toward IWLG expressed by Weibo users with various educational levels are *similar* across provinces, while the knowledge about IWLG *differs* significantly among Weibo users from different provinces. Similar to other studies (Zheng et al., 2022), we found that Weibo users held relatively inaccurate knowledge of IWLG at the provincial level (especially so for Qinghai, Ningxia, and Tibet), highlighting the variable effects of the Comprehensive Sexual Education (CSE) program in the Chinese mainland.

CSE is a curriculum-based teaching and learning process equipping children and youth to explore and learn the cognitive, affective, physical, and social aspects of sexuality (UN Women and UNICEF, 2018; Su et al., 2020). The provinces with higher education indices seem to emphasize the promotion of sexual education more in public schools. For example, in the Chinese mainland, the pioneers in implementing sexual education in primary and middle schools and embracing the advanced concepts of CSE are the provinces with higher education index ratings (e.g., Beijing, Shanghai; Liu and Su, 2014; UNESCO and UNFPA Offices in China, 2018). The outcomes yielded by education and sexual education thus appear to empower people to be more open to new ideas, discern authoritative and scientific information more thoroughly, facilitate more comprehensive knowledge, and be less likely to formulate misconceptions about sexual affairs, thus reducing prejudice toward IWLG significantly. This highlights the variable implementation of education and CSE programs in different provinces and the need for further research on their impact on attitudes toward IWLG.

Overall, the inclusion of provincial-level variables in our analysis based on the large-scale text sample represents a major point of difference from previous research on this topic (Chua et al., 2019; Wang et al., 2020; Zhang et al., 2020) and helps to provide a more nuanced understanding of the factors influencing attitudes toward IWLG in the Chinese mainland. These findings have important implications for policymakers and advocates seeking to promote acceptance and inclusion for sexual minority individuals in the Chinese mainland.

### 5.3. Implications

In line with the efforts to reduce stigma and discrimination against IWLG, our findings indicate that pursuing the following actions merit contemplation.

First, although China has admittedly undergone rapid growth and made extraordinary strides in economic development since the Reform and Opening-up, complex challenges remain with respect to achieving the Second Centenary Goals and realizing the Chinese Dream of national rejuvenation. One reality is that some western inland provinces lag behind coastal provinces and the rural–urban and regional divides continue to exist (United Nations Development Programme (UNDP), Tsinghua University, and State Information Center, 2019). Fortunately, the Chinese government is very concerned about this issue and is promoting it as a priority (The State Council Information Office of the People’s Republic of China, 2021). According to the modernization theory mentioned above, we predict that public opinion about IWLG will continue to shift to increased acceptance as a function of these efforts. Thus, we encourage any and all efforts to address any remaining imbalances and insufficient development with respect to residual IWLG prejudices among different provinces in China.

Second, the CSE has been confirmed to have a positive effect on individuals’ tolerance and acceptance of IWLG by various studies (Baams et al., 2017; Guo et al., 2019) and has been implemented in many countries (Liu and Su, 2014; Haberland and Rogow, 2015). Although more than 30 policies and documents published in the Chinese mainland have addressed sexuality education directly or indirectly (Chen, 2015), implementation of sexuality education in schools in China lags far behind this relatively progressive policy (Zhao et al., 2020). A recent report released by the UNESCO and UNFPA Offices in China (2018) has outlined many shortcomings regarding the implementation of sexuality education in middle schools in China. For example, few schools have a specific curriculum schedule or timetable for sexuality education; the education coverage rarely involves sexual orientation; and the teachers have a limited understanding of CSE and lack training and support. Many Chinese college textbooks still ignore the fact that CSSR was officially de-medicalized in 2001 and hold fast to the misconceptions that CSSR is a mental disorder and that individuals who are gay require conversion therapy (Parkin, 2018; Yang, 2019). Meanwhile, many parents, who are one of the most influential stakeholders to the CSE, have a limited understanding of CSE, and some of them even may oppose the implementation of CSE (Zhao et al., 2020). Given this state of affairs, more comprehensive policies or laws may well be needed to facilitate the sequential implementation of CSE to enhance its effectiveness. Establishment of a national mechanism to develop and review CSE textbooks is one approach worth considering to help ensure the scientific accuracy and authority of the content of CSE materials. Strengthening mainstream media coverage and popularization of CSE is another means to help aid the public to realize the importance and significance of CSE.

### 5.4. Limitations and suggestions for future research

It is important to note that this study has some limitations, which means that the findings should be interpreted with caution. One limitation is that the manual annotation process we utilized relied on others’ assessment rather than self-evaluation, which may misjudge the attitudes to some extent. Another limitation is that it was challenging to analyze and interpret how different variables interacted or changed over time at the provincial level, which led us to not include a time dimension in our study and potentially not fully utilize the data. Also, the supervised ML algorithms and TF-IDF we employed may have relatively lower performance compared to more advanced deep learning models (e.g., pre-trained language models). Future research could consider using more advanced deep learning models and semi-supervised ML or other methods to improve text classification performance and the efficiency and reliability of the manual annotation process. To improve the integrity of the data, it would be useful to use other keywords (e.g., English ones like “gay” and “lesbian”) to search and gather text data related to IWLG from Weibo. Additionally, it would be beneficial to analyze text data concerning IWLG from other online platforms in mainland China. Given the potential impact of the weighted indicators on the accuracy of our results, further optimization of these indicators, such as by considering additional factors or using more sophisticated weighting techniques, may be necessary in order to better reflect the attitudes toward IWLG in each province. Finally, it would be helpful to examine other provincial-level variables (e.g., cultural tightness, media exposure, collectivism, and individualism) that could potentially influence attitudes if reliable measures of these variables are available.

## 6. Conclusion

Our comprehensive search of Weibo text was designed to gain a more comprehensive understanding of the Chinese public’s perceptions of IWLG at the provincial level. This approach allowed us to expand the application scope of the modernization theory to cyberspace settings and revealed the potential value of incorporating data mining techniques (e.g., ML, NLP) for psychological research. In doing so, we found that the general attitude, feelings, and support for the equality of Weibo users toward IWLG among different provinces were relatively positive; meanwhile, the knowledge of Weibo users about IWLG was relatively inaccurate. Generally speaking, Weibo users in coastal provinces among Southeast China held more accepted attitudes toward IWLG than users in inland provinces. Economic development and educational level had significant positive effects on certain aspects of attitudes at the provincial level. Guided by our findings, several suggestions were offered to help further reduce and minimize public stigma, prejudice, and discrimination against IWLG.

## Code availability

The code that was used in this study is available from the corresponding author on request.

## Data availability statement

The original contributions presented in the study are included in the article/Supplementary material, further inquiries can be directed to the corresponding author.

## Ethics statement

Ethical review and approval was not required for the study on human participants in accordance with the local legislation and institutional requirements. Written informed consent from the participants’ legal guardian/next of kin was not required to participate in this study in accordance with the national legislation and the institutional requirements.

## Author contributions

QZ, YG, ZW, FA, ZK, SX, and XH contributed to the study conception and design and commented on previous versions of the manuscript. Material preparation, data collection, and analysis were performed by QZ, ZK, and SX. The first draft of the manuscript was written by QZ. All authors contributed to the article and approved the submitted version.

## Funding

This work was supported by the research funds from National Natural Science Foundation of China (grant number: 61937001).

## Conflict of interest

The authors declare that the research was conducted in the absence of any commercial or financial relationships that could be construed as a potential conflict of interest.

## Publisher’s note

All claims expressed in this article are solely those of the authors and do not necessarily represent those of their affiliated organizations, or those of the publisher, the editors and the reviewers. Any product that may be evaluated in this article, or claim that may be made by its manufacturer, is not guaranteed or endorsed by the publisher.

## Footnotes

^1^Although the term "homosexuality" was used in most of the articles we reference, it has become updated. In the current study, we, in line with Zheng et al. (2022), elect to use "consensual same-sex relations (CSSR)."

^2^For example, CSSR is one of the three main categories of sexual orientation and is considered a normal and natural variation in human sexuality, rather than a mental disorder.

^3^We used "31 provinces" to refer to the 31 provincial administrative divisions, which included 22 provinces, five autonomous regions, and four municipalities in the Chinese mainland, not including Hong Kong SAR, Macao SAR, and Taiwan.

^4^See https://github.com/fxsjy/jieba for details.

^5^According to the state’s "strategy for large-scale development of western China," the western region includes Chongqing, Gansu, Guangxi, Guizhou, Inner Mongolia, Ningxia, Qinghai, Shaanxi, Sichuan, Tibet, Xinjiang, and Yunnan. The eastern region includes Beijing, Fujian, Guangdong, Hebei, Jiangsu, Liaoning, Shandong, Shanghai, Tianjin, and Zhejiang. The central region includes Anhui, Heilongjiang, Henan, Hubei, Hunan, Jiangxi, Jilin, and Shanxi (Shen, 2004).

^6^This search index is provided by Baidu, one of the most popular and largest search engines in China. Based on the search data of Baidu users, the index uses data mining methods to analyze and cluster the attributes of people searching for the keyword, giving the distribution and ranking of the provinces to which the users belong. We obtained this keyword’s search index ranking of the 31 provinces in the Chinese mainland from July 1, 2013 (earliest available date) to December 31, 2020.
