# Supplementary material for: Status quo and predictors of Weibo users’ attitudes toward lesbians and gay men in 31 provinces in the Chinese mainland: Analysis based on supervised machine learning and provincial panel data
Source: Front Psychol. 2023 Feb 1;14:1069589. doi: 10.3389/fpsyg.2023.1069589 (PMC9931194; doi:10.3389/fpsyg.2023.1069589)
Supplement: Supplementary file 1 [file Table_1.DOCX]

Supplementary Material

# The data of Figure 2

| Provinces | Affect component | | | Behavior component | | | Cognition component | | | | General | | | |
| --- | --- | --- | --- | --- | --- | --- | --- | --- | --- | --- | --- | --- | --- | --- |
|  | Text size | Score  *M (SD)* | Adjust Score | Text size | Score  *M (SD)* | Adjust Score | Text size | Score  *M (SD)* | Adjust Score | | Text size | Score  *M (SD)* | Adjust Score | |
| ANHUI | 23054 | 3.39 (1.67) | 3.62 | 15581 | 3.27 (1.59) | 3.48 | 16252 | 2.64 (1.45) | | 2.81 | 18296 | 3.10 (1.57) | | 3.30 |
| BEIJING | 160153 | 3.28 (1.70) | 3.95 | 116640 | 3.29 (1.59) | 3.99 | 113231 | 2.78 (1.55) | | 3.36 | 130008 | 3.12 (1.61) | | 3.76 |
| CHONGQING | 23329 | 3.30 (1.69) | 3.45 | 16409 | 3.20 (1.63) | 3.34 | 15729 | 2.73 (1.50) | | 2.85 | 18489 | 3.08 (1.61) | | 3.21 |
| FUJIAN | 39491 | 3.28 (1.68) | 3.52 | 25983 | 3.25 (1.58) | 3.48 | 25992 | 2.66 (1.41) | | 2.85 | 30489 | 3.06 (1.56) | | 3.28 |
| GANSU | 6716 | 3.60 (1.64) | 3.68 | 4618 | 3.41 (1.57) | 3.49 | 5073 | 2.68 (1.41) | | 2.74 | 5469 | 3.23 (1.54) | | 3.30 |
| GUANGDONG | 162552 | 3.31 (1.68) | 4.05 | 111569 | 3.23 (1.59) | 3.94 | 113920 | 2.69 (1.48) | | 3.29 | 129347 | 3.08 (1.58) | | 3.76 |
| GUANGXI | 16430 | 3.35 (1.70) | 3.48 | 11409 | 3.25 (1.63) | 3.38 | 11301 | 2.73 (1.48) | | 2.84 | 13047 | 3.11 (1.60) | | 3.23 |
| GUIZHOU | 8355 | 3.61 (1.63) | 3.69 | 5737 | 3.45 (1.58) | 3.53 | 6068 | 2.74 (1.46) | | 2.81 | 6720 | 3.27 (1.56) | | 3.34 |
| HAINAN | 7305 | 3.27 (1.74) | 3.32 | 5235 | 3.07 (1.67) | 3.12 | 4951 | 2.57 (1.41) | | 2.61 | 5830 | 2.97 (1.61) | | 3.02 |
| HEBEI | 21926 | 3.45 (1.68) | 3.70 | 15920 | 3.36 (1.60) | 3.60 | 15200 | 2.86 (1.52) | | 3.06 | 17682 | 3.22 (1.60) | | 3.45 |
| HENAN | 26141 | 3.49 (1.66) | 3.78 | 18176 | 3.38 (1.59) | 3.66 | 18428 | 2.82 (1.51) | | 3.06 | 20915 | 3.23 (1.59) | | 3.50 |
| HEILONGJIANG | 14115 | 3.42 (1.70) | 3.57 | 10267 | 3.29 (1.64) | 3.44 | 9847 | 2.77 (1.51) | | 2.89 | 11410 | 3.16 (1.62) | | 3.30 |
| HUBEI | 29638 | 3.43 (1.69) | 3.68 | 21266 | 3.35 (1.61) | 3.60 | 20428 | 2.81 (1.52) | | 3.01 | 23777 | 3.20 (1.61) | | 3.43 |
| HUNAN | 24945 | 3.35 (1.69) | 3.57 | 17102 | 3.27 (1.60) | 3.48 | 16578 | 2.71 (1.51) | | 2.89 | 19542 | 3.11 (1.60) | | 3.31 |
| JILIN | 12029 | 3.42 (1.69) | 3.53 | 8300 | 3.23 (1.64) | 3.33 | 8531 | 2.72 (1.45) | | 2.81 | 9620 | 3.12 (1.59) | | 3.22 |
| JIANGSU | 57282 | 3.33 (1.69) | 3.71 | 40220 | 3.27 (1.60) | 3.65 | 40609 | 2.77 (1.51) | | 3.10 | 46037 | 3.12 (1.60) | | 3.49 |
| JIANGXI | 14294 | 3.37 (1.71) | 3.51 | 10053 | 3.26 (1.64) | 3.39 | 9852 | 2.75 (1.49) | | 2.86 | 11400 | 3.13 (1.61) | | 3.26 |
| LIAONING | 25434 | 3.38 (1.70) | 3.62 | 18079 | 3.27 (1.61) | 3.50 | 17229 | 2.77 (1.52) | | 2.96 | 20247 | 3.14 (1.61) | | 3.36 |
| INNER MONGOLIA | 8858 | 3.56 (1.64) | 3.66 | 6095 | 3.43 (1.57) | 3.52 | 6491 | 2.74 (1.46) | | 2.81 | 7148 | 3.24 (1.56) | | 3.33 |
| NINGXIA | 4535 | 3.60 (1.62) | 3.63 | 2984 | 3.37 (1.56) | 3.40 | 3475 | 2.51 (1.31) | | 2.53 | 3665 | 3.16 (1.50) | | 3.19 |
| QINGHAI | 3802 | 3.65 (1.60) | 3.69 | 2467 | 3.39 (1.56) | 3.43 | 3065 | 2.49 (1.29) | | 2.52 | 3111 | 3.18 (1.48) | | 3.21 |
| SHANDONG | 45329 | 3.43 (1.67) | 3.78 | 31043 | 3.30 (1.61) | 3.63 | 31253 | 2.78 (1.51) | | 3.06 | 35875 | 3.17 (1.59) | | 3.49 |
| SHANXI | 11547 | 3.53 (1.66) | 3.70 | 8106 | 3.44 (1.57) | 3.60 | 8427 | 2.76 (1.48) | | 2.89 | 9360 | 3.24 (1.57) | | 3.40 |
| SHAANXI | 17869 | 3.49 (1.66) | 3.69 | 12496 | 3.40 (1.59) | 3.59 | 12455 | 2.74 (1.50) | | 2.89 | 14273 | 3.21 (1.58) | | 3.39 |
| SHANGHAI | 85523 | 3.36 (1.67) | 3.75 | 64849 | 3.34 (1.58) | 3.75 | 59725 | 2.75 (1.56) | | 3.07 | 70032 | 3.15 (1.61) | | 3.52 |
| SICHUAN | 74261 | 3.38 (1.65) | 3.82 | 49583 | 3.27 (1.62) | 3.68 | 51324 | 2.66 (1.41) | | 2.99 | 58389 | 3.10 (1.56) | | 3.50 |
| TIANJIN | 14759 | 3.24 (1.71) | 3.33 | 10619 | 3.13 (1.62) | 3.21 | 10325 | 2.71 (1.49) | | 2.78 | 11901 | 3.02 (1.61) | | 3.11 |
| TIBET | 3714 | 3.55 (1.66) | 3.57 | 2478 | 3.36 (1.57) | 3.37 | 2893 | 2.58 (1.34) | | 2.60 | 3028 | 3.16 (1.52) | | 3.18 |
| XINJIANG | 7238 | 3.51 (1.64) | 3.57 | 4943 | 3.43 (1.56) | 3.49 | 5231 | 2.62 (1.39) | | 2.66 | 5804 | 3.18 (1.53) | | 3.24 |
| YUNNAN | 12593 | 3.39 (1.72) | 3.53 | 9042 | 3.21 (1.62) | 3.34 | 8532 | 2.66 (1.47) | | 2.77 | 10056 | 3.09 (1.60) | | 3.21 |
| ZHEJIANG | 54509 | 3.35 (1.67) | 3.70 | 37685 | 3.27 (1.58) | 3.61 | 38943 | 2.69 (1.47) | | 2.98 | 43712 | 3.10 (1.57) | | 3.43 |
